# Real-world survival outcomes of neoadjuvant versus adjuvant chemotherapy in operable triple-negative breast cancer: a propensity score matched registry-based study

**DOI:** 10.2340/1651-226X.2025.43990

**Published:** 2025-10-01

**Authors:** Ali Inan El-Naggar, Andreas Karakatsanis, Antonios Valachis

**Affiliations:** a Department of Oncology, Faculty of Medicine and Health, Örebro University, Örebro, Sweden; b Department of Surgical Sciences, Uppsala University Hospital, Uppsala University, Uppsala, Sweden; c Section for Breast Surgery, Department of Surgery, Uppsala University Hospital, Uppsala, Sweden

**Keywords:** Triple negative breast neoplasms, neoadjuvant therapy, adjuvant chemotherapy, treatment outcome

## Abstract

**Background and purpose:**

Triple-negative breast cancer (TNBC) is an aggressive breast cancer subtype where the prognostic implications of primary systemic therapy followed by surgery, compared to up-front surgery and subsequent adjuvant chemotherapy (ACT), are yet to be outlined. This retrospective registry-based study aimed to compare survival outcomes between patients treated with neoadjuvant chemotherapy (NACT) versus ACT for operable TNBC in a real-world setting.

**Patient/material and methods:**

We included all patients treated with chemotherapy for operable TNBC in Sweden between 2008 and 2019 using the Swedish national research database BCBaSe 3.0. To reduce confounding by indication, we implemented propensity score matching (PSM) and main study outcomes were defined as distant disease-free survival (DDFS), breast cancer-specific survival (BCSS) and overall survival (OS).

**Results:**

A total of 4,704 patients were included in the study, of which 1,183 received NACT. Following 1:1 PSM, 837 patients in each treatment setting were available for analyses. We found no statistically significant differences in terms of DDFS (adjusted hazard ratio [aHR] 1.18; 95% confidence interval [CI] 0.93 – 1.50), BCSS (aHR 1.10; 95% CI 0.83 – 1.45) or OS (aHR 1.07; 95% CI 0.82 – 1.39) between patients treated with NACT versus ACT. However, subgroup analysis of patients with clinically node-positive disease (cN+) demonstrated a significant DDFS benefit of NACT (aHR 0.65; 95% CI 0.47 – 0.90).

**Interpretation:**

Overall, we found comparable survival among patients with TNBC treated with NACT or ACT. Considering the anticipated survival improvements when response-guided post-neoadjuvant strategies are implemented in clinical practice, our findings may support the use of NACT in operable TNBC.

## Introduction

Triple-negative breast cancer (TNBC) accounts for 12–17% of all breast cancers in women and is associated with an intrinsically aggressive behaviour, high rates of recurrence and impaired survival outcomes [1]. Current international guidelines recommend primary systemic treatment with neoadjuvant chemotherapy (NACT) in patients with operable TNBC when the tumour is larger than 10 mm (at least T1c) and/or in clinically node-positive disease [2–4]. This indicates a transition in the treatment sequence of curative intent from adjuvant chemotherapy (ACT) in the postoperative setting which has been the mainstay of care for several decades [5, 6]. NACT in primarily operable breast cancers enables an *in vivo* response evaluation of initiated therapies, increases the likelihood of breast-conserving surgery and aims to eradicate undetectable systemic dissemination early on [2, 3, 7]. Furthermore, pathological complete response (pCR) to NACT has demonstrated prognostic value and post-neoadjuvant strategies based on the results of preoperative treatment provide opportunities for individualised therapeutic (de-)escalation [8–11]. Meanwhile, intensification of neoadjuvant therapies is generally associated with additional clinical and radiological follow-up before surgery, which could prove challenging in the setting of limited healthcare resources. Despite the potential advantages of NACT in operable TNBC, a survival benefit of NACT in direct comparison with ACT has not been demonstrated, thus causing debate on the most appropriate treatment strategy [12]. The aim of this registry-based study was to investigate the potential benefits of using NACT compared to ACT with regards to distant disease-free survival (DDFS), breast cancer-specific survival (BCSS) and overall survival (OS) among Swedish patients with operable TNBC.

## Patients/material and methods

The study was reported using the ESMO Guidance for Reporting Oncology Real-World Evidence (ESMO-GROW) [13].

### Study design and data sources

We performed a retrospective, registry-based and propensity score matched (PSM) study using the research database BCBaSe 3.0 with coverage of nearly all patients with recently diagnosed breast cancer in Sweden from 2008 to 2019. BCBaSe 3.0 provides data from the Swedish National Quality Registry of Breast Cancer (NKBC) regarding approximately 115,000 individuals with breast cancer and is linked to data from several other national registries based on individual social security numbers. For the purpose of this study, data from the NKBC (on tumour characteristics, treatment modalities and disease recurrence), the National Cause of Death Register (time and cause of death) and the Longitudinal Integrated Database for Health Insurance and Labour Market Studies (LISA, socioeconomic aspects) were collected and used for statistical analyses.

### Study cohort

All patients diagnosed with operable TNBC who were treated with curative intent using either NACT or ACT, between January 2008 and December 2019, were included in the study. TNBC was defined as ER and PR < 10% by immunohistochemistry and negative HER2-status in accordance with the ASCO-CAP guidelines [14]. Patients with tumours expressing ER 1–9% (ER-low) were included as a subgroup of special interest due to historical discrepancies in the classification of truly ER-negative disease (ER < 1% vs. ER-low) [15]. The study definition of TNBC as ER < 10% was applied based on the growing body of evidence suggesting that ER-low tumours share biological and clinical characteristics more similar to ER-negative (ER < 1%) breast cancers than their ER-positive counterparts [16, 17]. Patients with de novo metastatic disease (M1; defined as the presence of metastasis within 3 months from breast cancer diagnosis), inflammatory breast cancer or inoperable tumours due to locoregional extent (cT4 and/or cN3) were excluded as preoperative treatment is required to enable curative surgery.

### Study outcomes and definitions

The main study outcomes were defined as the time-to-event endpoints DDFS, BCSS and OS as summarised by the DATECAN initiative 2015 [18]. Hence, DDFS was defined as the time from diagnosis of breast cancer until distant metastasis or death by any cause. BCSS was defined as the time from diagnosis of breast cancer until death due to breast cancer and OS was defined as the time from diagnosis of breast cancer until death by any cause. Pathological complete response (pCR) after NACT was defined as the absence of invasive disease in the breast and axilla (ypT0/is ypN0).

### Statistical analyses

Comparisons in baseline characteristics between the two treatment groups (NACT vs. ACT) were performed using the chi-square test or Wilcoxon Rank Sum test, as appropriate. To enable justifiable comparisons of outcomes and mitigate the risk of confounding by indication – that is, the inherent bias of choosing NACT for patients with more advanced or high-grade breast cancers, the propensity score matched (PSM) approach was used. Logistic regression model was applied to estimate propensity scores, taking into consideration patient- and tumour-related characteristics that could potentially influence treatment decisions (age, income, region of residence, cT, cN and ER-status). Propensity scores were then matched using a 1:1 nearest-neighbour matching method and the post-PSM baseline characteristics for the two groups (NACT vs. ACT) were comparatively re-evaluated using chi-square test or Wilcoxon Rank Sum test*.* Standardised differences (SD) < 0.1 (with respect to each variable) were considered successful propensity score matching (PSM).

Survival outcomes (DDFS, BCSS and OS) were analysed using multivariable Cox proportional hazards regression analyses to calculate hazard ratios (HRs) and 95% confidence intervals (CI), using the PSM populations and illustrated by Kaplan–Meier curves. HRs were adjusted for age, cT, cN, region of residence, income, estrogen receptor status, type of surgical intervention in breast and axilla as well as postoperative radiotherapy. Two sensitivity analyses were performed with multivariable Cox proportional hazards regression. One was restricted to DDFS exclusively in patients from the region of Stockholm-Gotland, a health care region where data reports on distant recurrence are considerably more comprehensive than in the Swedish NKBC overall. A second sensitivity analysis was performed in the pre-PSM population by utilising propensity scores for calculating inverse-probability of treatment-weights (IPTW; defined as the inverse probability of patients to receive the treatment they actually received) to enable the inclusion of all patients in the IPTW-adjusted models.

Three subgroups of clinical interest where the comparisons of NACT versus ACT could be especially valuable were identified and subgroup analyses were performed in the PSM populations for patients with clinically node-positive disease (cN+), clinically node-negative disease (cN0) and ER < 1%. Cox proportional hazards regression models were used in each subgroup analysis for the respective survival outcomes.

Rates of pCR were estimated in the NACT group for both the pre-PSM and PSM cohort and presented with 95% CIs.

All multivariable analyses were complete case analysis without any imputation strategy for missing values. All p-values in the analyses were two-sided and the threshold for statistical significance was set at < 0.05. Adjusted HRs were presented with corresponding 95% CIs. Statistical analyses were performed using SPSS version 28.0 (IBM SPSS Statistics for Windows, Version 28.0. Armonk, NY: IBM Corp).

## Results

### Study cohort characteristics

**Figure 1:**
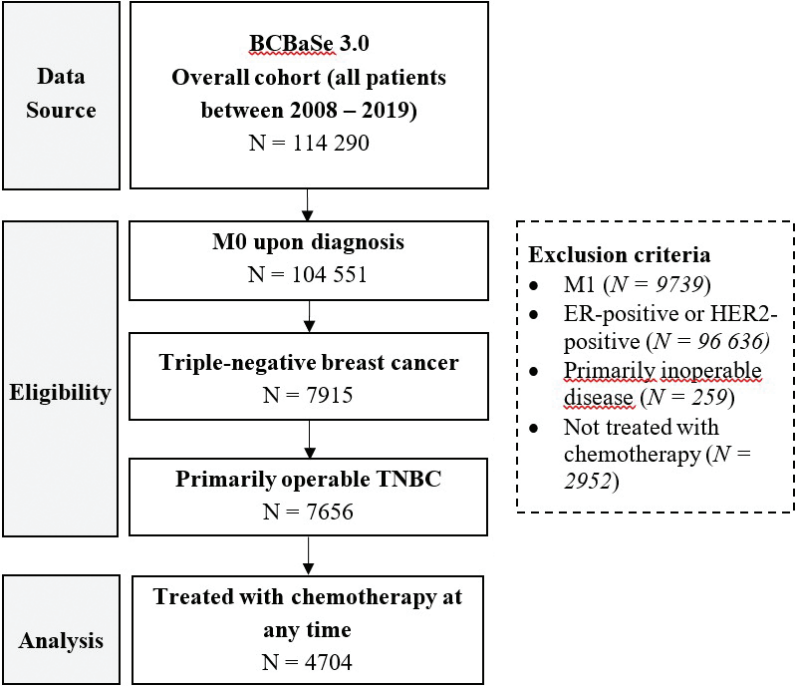
Flowchart diagram of case selection process for analysis.

The patient selection process aligned with previously described inclusion and exclusion criteria resulted in 4,704 eligible patients in the study cohort, out of which 1,183 received NACT and 3,521 were treated with ACT (Figure 1). Baseline characteristics in the treatment groups before PSM are described in Table 1. Patients treated with NACT were generally younger, had more advanced tumours (> cT1 and/or cN+) and were more likely to undergo extensive surgery (mastectomy and/or axillary lymph node dissection [ALND]) as well as postoperative radiotherapy involving regional lymph nodes. Some regional differences and differences related to socioeconomic factors were also observed. A higher proportion of patients treated with NACT had missing values for histological subtype, tumour grade and immunohistochemical proliferation index (Ki-67*)*.

**Table 1:** Baseline characteristics between treatment groups before propensity score matching.

Characteristics	Neoadjuvant chemotherapy *N* = 1,183; *n* (%)	Adjuvant chemotherapy *N* = 3,521; *n* (%)	*P*
Age, median (Q1–Q3)	52 (41–62)	59 (48–67)	<0.001
Household income			
Quartile 1 (lowest)	183 (15.6)	664 (19.0)	<0.001
Quartile 2	264 (22.5)	850 (24.3)	
Quartile 3	301 (25.6)	1,031 (29.5)	
Quartile 4 (highest)	427 (36.3)	954 (27.3)	
Region of residence			
North	73 (6.2)	342 (9.7)	<0.001
Stockholm-Gotland	412 (35.0)	640 (18.2)	
Uppsala-Örebro	121 (10.3)	692 (19.7)	
South	250 (21.2)	512 (14.6)	
Southeast	110 (9.3)	402 (11.5)	
West	211 (17.9)	922 (26.3)	
cT			
T1	198 (16.7)	2,147 (61.0)	<0.001
T2 or T3	985 (83.3)	1,374 (39.0)	
cN			
N0	636 (53.8)	3,074 (87.3)	<0.001
N1 or N2	547 (46.2)	447 (12.7)	
Histology			
Ductal	379 (32.0)	3,005 (85.3)	0.001
Lobular	15 (1.3)	48 (1.4)	
Other	39 (3.3)	441 (12.5)	
Missing	750 (63.4)	27 (0.8)	
ER-status			
ER < 1%	1,147 (97.0)	3,276 (93.0)	<0.001
ER low positive	36 (3.0)	245 (7.0)	
Tumor grade			
I	8 (0.7)	33 (0.9)	<0.001
II	153 (12.9)	480 (13.6)	
III	274 (23.2)	2,950 (83.8)	
Missing	750 (63.4)	58 (16.5)	
Ki-67, median (Q1-Q3)	59.5 (20 – 80)	68 (43 – 82)	<0.001
Missing	735 (62.1)	980 (27.8)	
Surgery of primary tumor			
Breast conserving surgery	479 (41.8)	2,222 (63.1)	<0.001
Mastectomy	667 (58.2)	1,298 (36.9)	
Axillary surgery			
SLND	387 (34.4)	2,517 (72.9)	<0.001
ALND	588 (52.3)	493 (14.3)	
SLND => ALND	150 (13.3)	444 (12.9)	
Radiation therapy			
No	258 (21.8)	853 (24.2)	<0.001
Yes, breast or chest wall	327 (27.6)	1,852 (52.6)	
Yes, breast and lymph nodes	598 (50.5)	816 (23.2)	

cT: clinical tumor status; cN: clinical node status; ER: estrogen receptor; SLND: sentinel lymph node dissection; ALND: axillary lymph node dissection.

### Study cohort after propensity-score matching

Subsequent 1:1 PSM resulted in a total of 837 patients in the NACT and ACT groups respectively. Baseline characteristics in the two groups after PSM are described in Table 2 and were essentially well-balanced, corresponding to successful matching. The variables with remaining statistically significant differences between the two treatment groups after PSM were related to the extent of axillary surgery and the target of adjuvant radiotherapy.

**Table 2:** Baseline characteristics between treatment groups after propensity score matching (variables included in the propensity score were age, income, region of residence, cT, cN, ER-status).

Characteristics	Neoadjuvant chemotherapy *N* = 837; *n* (%)	Adjuvant chemotherapy *N* = 837; *n* (%)	*P*
Age, median (Q1–Q3)	54 (43–64)	54 (44–64)	0.807
Household income			
Quartile 1 (lowest)	142 (17.0)	137 (16.4)	0.801
Quartile 2	192 (22.9)	206 (24.6)	
Quartile 3	222 (26.5)	209 (25.0)	
Quartile 4 (highest)	281 (33.6)	285 (34.1)	
Region of residence			
North	62 (7.4)	65 (7.8)	0.880
Stockholm – Gotland	244 (29.2)	238 (28.4)	
Uppsala-Örebro	104 (12.4)	95 (11.4)	
South	166 (19.8)	158 (18.9)	
Southeast	92 (11.0)	93 (11.1)	
West	169 (20.2)	188 (22.5)	
cT			
T1	190 (22.7)	175 (20.9)	0.375
T2 or T3	647 (77.3)	662 (79.1)	
cN			
N0	553 (66.1)	564 (67.4)	0.568
N1 or N2	284 (33.9)	273 (32.6)	
ER-status			
ER < 1%	803 (95.9)	808 (96.5)	0.521
ER low positive	34 (4.1)	29 (3.5)	
Surgery of primary tumour			
Breast conserving surgery	363 (44.8)	407 (48.6)	0.121
Mastectomy	447 (55.2)	430 (51.4)	
Axillary surgery			
SLND	330 (41.6)	458 (55.6)	<0.001
ALND	361 (45.5)	252 (30.6)	
SLND => ALND	102 (12.9)	114 (13.8)	
Radiation therapy			
No	199 (23.8)	204 (24.4)	0.005
Yes, breast or chest wall	262 (31.3)	317 (37.9)	
Yes, breast and lymph nodes	376 (44.9)	316 (37.8)	

### Rates of pCR

The pCR rates in patients treated with NACT were 44.4 % (95% CI: 41.6–47.2%) and 43.2% (95% CI: 39.9–46.6%) in the pre-PSM and PSM cohorts respectively.

### Survival outcomes in patients treated with NACT versus ACT

**Figure 2:**
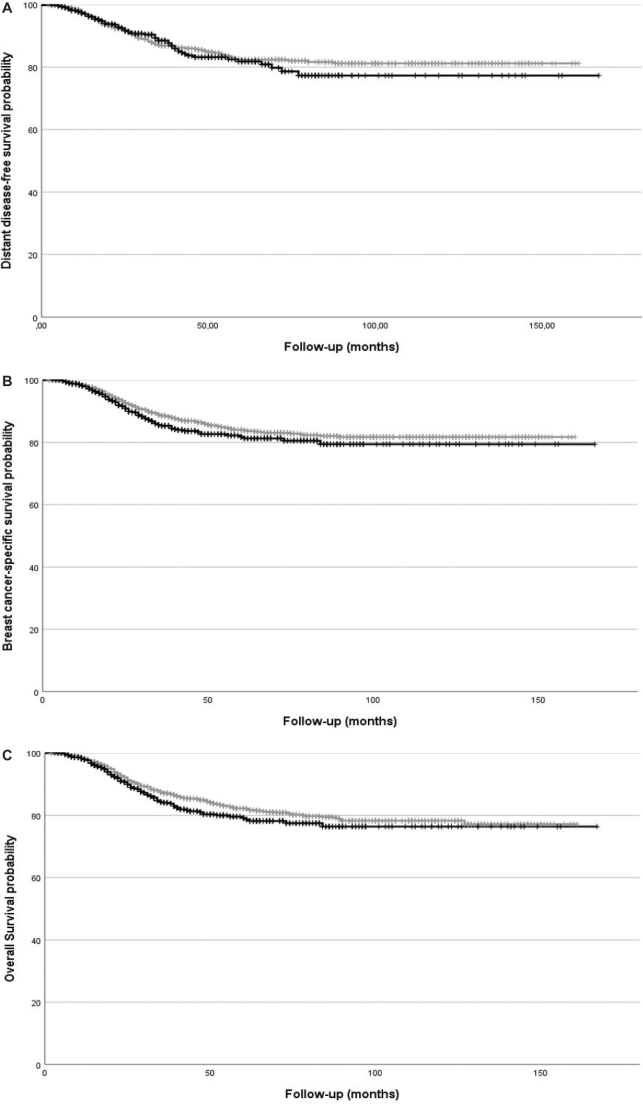
Kaplan–Meier curves for survival outcomes by treatment group in propensity score matching cohort: (a) Distant disease-free survival; (b) Breast cancer-specific survival; (c) Overall survival. Curves with grey line represent patients treated with adjuvant therapy whereas curves with black line represent patients treated with neoadjuvant therapy.

Analyses of survival outcomes in the PSM cohort showed no statistically significant differences in any measured endpoint between treatment with NACT and ACT (Table 3 and illustrated by Kaplan–Meier curves in Figure 2). This was also evident in the sensitivity analysis using the pre-PSM cohort while including IPTW in the adjusted models, as well as the DDFS analysis when limited to patients from the region of Stockholm-Gotland.

**Table 3:** Multivariable Cox proportional hazards regression analyses for survival outcomes between treatment groups (adjuvant vs. neoadjuvant) after propensity score matching including sensitivity analyses.

Outcome	Adjusted hazard ratio^a^	95% confidence interval
Adjuvant versus neoadjuvant chemotherapy		
Distant disease-free survival (PSM cohort)	1.18	0.93–1.50
Breast cancer-specific survival (PSM cohort)	1.10	0.83–1.45
Overall survival (PSM cohort)	1.07	0.82–1.39
Distant disease-free survival (PSM cohort; sensitivity analysis including only patients from Stockholm-Gotland)	1.00	0.61–1.64
Distant disease-free survival (sensitivity analysis^b^)	1.17	0.96–1.43
Breast cancer-specific survival (sensitivity analysis^b^)	1.11	0.88–1.40
Overall survival (sensitivity analysis^b^)	1.10	0.89–1.37

PSM: propensity score matching.

a Adjusted for age, cT, cN, region, income, estrogen-receptor status, surgery type in breast and axilla, postoperative radiation therapy.

b Sensitivity analysis using the pre-propensity score matching (PSM) cohort and including IPTW into the adjusted models. Same variables as above were used as covariates.

### Survival outcomes in subgroups

Subgroup analyses of survival outcomes after PSM in patients with clinically node-negative disease (*N* = 1,117) and tumours with ER < 1% (*N* = 1,611) showed no statistically significant differences between treatment groups respectively (Table 4). Patients with clinically node-positive disease (*N* = 557) who received NACT were less likely to be affected by distant relapse compared to the ACT group (aHR 0.65, 95% CI 0.47–0.90), with a non-statistical significance in terms of BCSS and OS.

**Table 4:** Subgroup analyses for survival outcomes between treatment groups (adjuvant vs. neoadjuvant) after propensity score matching.

Outcome	Adjusted hazard ratio	95% confidence interval
Adjuvant versus neoadjuvant chemotherapy		
cN+ (*N* = 557)^a^		
Distant disease-free survival	0.65	0.47–0.90
Breast cancer-specific survival	0.69	0.47–1.01
Overall survival	0.72	0.51–2.07
cN0 (*N* = 1,117)^a^		
Distant disease-free survival	1.21	0.85–2.24
Breast cancer-specific survival	1.33	0.86–2.06
Overall survival	1.30	0.87–1.95
ER < 1% only (*N* = 1,611)^b^		
Distant disease-free survival	1.22	0.97–1.56
Breast cancer-specific survival	1.13	0.85–1.50
Overall survival	1.09	0.84–1.43

PSM: propensity score matching.

a Adjusted for age, cT, region, income, estrogen-receptor status, surgery type in breast and axilla, postoperative radiation therapy.

b Adjusted for age, cT, cN, region, income, surgery type in breast and axilla, postoperative radiation therapy.

## Discussion and conclusion

In this retrospective cohort study of more than 4,500 patients with operable TNBC, 1:1 PSM resulted in 837 patients treated with NACT and ACT, respectively. We found no statistically significant differences in survival outcomes between the two treatment strategies whereas subgroup analyses identified a benefit of NACT in patients with cN+ disease in terms of DDFS.

A meta-analysis by the Early Breast Cancer Trialists’ Collaborative Group (EBCTCG) investigated long-term benefits and risks of NACT compared to ACT using individual patient data from 4,756 patients with breast cancer included in 10 randomised trials, suggesting no survival benefits of NACT in the subgroup of patients with ER- and PR-negative tumours [12]. However, there was no information on HER2-status which is now regarded as part of routine testing due to its predictive and prognostic importance.

With focus on triple-negative disease, a meta-analysis by Xia et al. compared survival outcomes of NACT versus ACT among more than 35,000 patients with TNBC [19]. They found no statistically significant differences in disease-free survival (DFS) between the two treatment strategies but identified poorer OS with NACT compared to ACT (HR 1.59, 95% CI 1.25–2.02) – a finding inconsistent with our study results, where OS was found to be comparable between the two groups. An important remark is that most studies included in the meta-analysis were observational, of retrospective design and unlike our study in which both PSM-matching and IPTW-adjusted methods were applied, there were no suitable statistical approaches to mitigate the high risk of indication bias. This source of bias is of particular importance considering how NACT has historically been the preferred strategy for patients with more aggressive tumours or advanced disease presentation. In such clinical settings, the choice of NACT may rather serve as a proxy for impaired survival due to up-front unfavourable tumour biology, which renders outcome comparisons between NACT and ACT challenging. It has already been confirmed that patient- and tumour-related aspects such as age, tumour size and nodal involvement, influence the decision of whether to initiate NACT or to opt for primary surgery followed by chemotherapy in the adjuvant setting [20]. Such a relationship was also evident in our study, as patients with more advanced disease were more likely to receive NACT. This emphasises the necessity for appropriate statistical approaches, such as PSM, to enable meaningful comparisons of the two treatment strategies in a real-world setting. Our use of a PSM methodology is an effort to mitigate this risk of indication bias and hence provides more reliable results.

Results from the same meta-analysis led to the conclusion that patients who achieved pCR from NACT (with an average pCR rate of 35%) had significantly better OS and DFS than patients in the ACT group [19]*.* This suggests that the supposedly detrimental effect of NACT identified in their pooled analysis, which was not the case in our cohort, can be explained at least in part by patients with residual disease. Although the pCR rates from our cohort indicate that treatment regimens used in the neoadjuvant setting were adequate, NACT in TNBC can be further optimised by the addition of a platinum salt and immune checkpoint inhibitor [21, 22]. However, these treatment strategies were not used in our study cohort as the evidence on the role of checkpoint inhibitors and platinum salts in the neoadjuvant setting in terms of improved recurrence-free survival was published after 2019. Considering the prognostic role of pCR in TNBC [8, 9], it is likely that further improvement in pCR rates would result in better survival outcomes for patients treated with NACT.

Contemporary guidelines recommend NACT based not only on the increased frequency of breast-conserving surgery but also on the prognostic value of achieving pCR as well as the prospect of adopting so called post-neoadjuvant strategies for patients with residual disease [2, 3]. Currently, the addition of perioperative immunotherapy to NACT has become standard of care for patients with triple-negative tumours >2 cm and/or cN+ [22]. Post-neoadjuvant capecitabine for those with residual disease after NACT is another treatment escalation that has demonstrated survival benefit and the same has been established for (post-neo)adjuvant PARP-inhibitors in patients with germline pathogenic variants of BRCA1/2 – a genotype particularly associated with the development of TNBC [10, 11]. In the present study, survival outcomes of patients treated with NACT were equivalent to those receiving ACT. The lack of statistically convincing benefit of NACT (apart from cN+ disease where DDFS was improved), despite primary systemic treatment being regarded as modern standard, should be interpreted in the absence of these aforementioned novel post-neoadjuvant therapeutic strategies. That is, patients in our study did not receive immune checkpoint inhibitors as part of their perioperative therapy, nor was response-guided post-neoadjuvant therapies used for non-responders. Our cohort was treated prior to the implementation of these therapies and therefore, postoperative regimens did not reflect current guideline-based practice. One could hypothesise that the adoption of such therapeutic strategies in our study population would have had a positive impact in the long-term outcomes of the patients treated with NACT. This may be especially true for the addition of immune checkpoint inhibitors which have now become standard of care but only in the preoperative setting. Use of these drugs with tumour *in loco* allows for a presumably beneficial interplay between tumour antigens and immune cells with a potential to enhance anti-tumoral efficacy. The clinical impact of this has been successfully investigated in neoadjuvant treatment of melanoma [23, 24]. Such an interaction is not possible after primary surgery, additionally supporting the neoadjuvant approach.

The fact that patients with cN+ disease derived greater benefit from NACT emphasises that nodal metastasis upon initial presentation is a marker of more aggressive and extensive disease where early initiation of systemic therapy to eradicate micrometastatic dissemination seems to be of substantial importance. This finding may, therefore, reflect the shortened time interval from diagnosis of TNBC to start of systemic therapy in the neoadjuvant approach as compared to the adjuvant setting. Time from diagnosis to systemic therapy and its association to prognosis is evident in the adjuvant setting as well, where longer time intervals seem to compromise survival [25, 26].

In the pre-PSM cohort, mastectomy and ALND were more common among patients treated with NACT. After PSM, mastectomy rates were similar, suggesting an improvement in breast surgical de-escalation. However, ALND remained more frequent in the NACT group, indicating that its potential benefit in N+ disease has not yet translated into de-escalation of axillary surgery. Further refinements in surgical approaches are needed, especially with emerging strategies to safely reduce the extent of axillary interventions in cN+ patients responding well to NACT [27–30]. Additionally, less extensive surgery may lower the rate of postoperative complications and recovery delays, minimising disruptions in the start of postoperative therapies [31].

In terms of study limitations, we must acknowledge the inherent flaws and bias in the retrospective nature of this study and that propensity score-based statistical approaches, although suitable, cannot rule out all sources of indication bias. As a result, a risk of residual bias that could influence treatment decisions on NACT versus ACT and patient outcomes is still present. Our study demonstrates several strengths as it includes a large cohort of patients with breast cancer from a high-quality nation-wide database with direct linkage to several population-based registries, covering both tumour-related and socioeconomic factors. We applied various statistical approaches such as PSM and IPTW adjustments to mitigate the impact of indication bias.

In summary, we found comparable survival among patients with TNBC treated with NACT versus ACT, except in patients with cN+ disease where NACT seems to offer some benefit. Considering the anticipated survival improvement when response-guided post-neoadjuvant strategies are implemented in clinical practice, our results may support the use of NACT in operable TNBC as a preferred therapeutic approach.

## Data Availability

The datasets analysed in the current study are not publicly available due to data protection rules for sensitive (pseudonymised) data but are available from the corresponding author on reasonable request.
